# Measuring Health Equity for Ambulatory Care Sensitive Conditions in a Large Integrated Health Care System: The Development of an Index

**DOI:** 10.1089/heq.2018.0092

**Published:** 2019-04-03

**Authors:** Alice Pressman, Stephen Lockhart, John Petersen, Sarah Robinson, Maria Moreno, Kristen M.J. Azar

**Affiliations:** ^1^ Sutter Health Research Enterprise, Center for Health Systems Research, Walnut Creek, California.; ^2^ Sutter Health Quality Improvement, Office of Patient Experience, Sacramento, California.

**Keywords:** ambulatory care sensitive conditions, health disparities, health equity, quality improvement

## Abstract

Disparities in outcomes for preventive and primary health care services often result when vulnerable patients rely on episodic encounters for emergency services that do not meet their long-term health needs. Understanding health outcomes in socially or economically disadvantaged subgroups is crucial to improving community health, and it requires innovative analytics and dynamic application of clinical and population data. While it is common practice to use proxy indicators, such as quality of life and mortality, when discussing health equity, these have shown limited utility and are rarely applied at a population-level within a health system. Therefore, we designed and implemented an index, calculated as the ratio of observed-to-expected encounters, to identify and quantify health inequalities in health care systems. Providing equitable care, as measured by health outcomes, is analogous to precision medicine applied to social determinants. For health systems, the use of this index will facilitate the development of specially-tailored interventions to address inequity and provides a tool to measure the impact of such programs.

## Introduction

Addressing health inequity, or differences in health outcomes between some population subgroups,^1^ requires innovative analytics and the dynamic application of health and population data to accurately identify inequity in real time. Recently, the National Institutes of Health called for an enhancement in their capacity to address health disparities in its 2009–2013 Strategic Plan^2^ and articulated a need for research aimed at furthering the evolution of health disparities research from its predominantly descriptive methods of identifying and addressing health care inequities, to analytic and actionable approaches through the increased application of advanced health information technology and tools. Despite this call to action, little progress has been made in part because of the difficulty in measuring and monitoring changes in inequities within health systems in real time.^3–6^

To date, proxy indicators to measure and inform health disparities, such as quality of life and mortality, have shown limited utility and have rarely been implemented at a population level within a health system dashboard.^7^ A commonly used indicator of disadvantage and inequity has been disproportionate absolute numbers of hospital admissions for ambulatory care sensitive conditions (ACSCs),^8–10^ and, as a result, health systems have begun to monitor and report this activity. It is recognized that, effectively managed, these conditions can be treated in an outpatient setting given the appropriate access to quality care, reducing the incidence of acute illness and hospitalization.^11^ Although previous studies have used electronic health record (EHR) data to measure equity through volume of ACSCs, there remains a large need to develop rigorous and widely applicable tools to quantify inequity in a health system, accounting for demographic variation existing in the area of service on a large system-wide scale.^12–16^ Given the wealth of health care data now available for large health care systems, there is tremendous potential to harness and synthesize a variety of data elements in addition to EHR information on clinical encounters. These include, but are not limited to, event and disease registries, health system utilization, cost information, disease status, and quality and safety data.^7^ Applying analytics, which combine existing health system data with population data, offers a unique opportunity to identify and address disparities with greater precision because the data are available in real time, are objectively collected within the system, and are already being captured through systematic clinical and administrative protocols during the patient experience.

Large health systems provide a window into a representative segment of the population within regions of the country and, therefore, have the potential to enhance our understanding of disparities and to advance our efforts to achieve health equity in new and innovative ways. Sutter Health, a large integrated health system in Northern California, is one such example. Serving >3 million patients in the most diverse state in the nation in both urban and rural settings, Sutter provides a unique opportunity to investigate new and innovative solutions. To address the existing lack of real-time dynamic measurement solutions for health equity, we used a novel empirical approach to design and implement an index to identify and prioritize health inequities for ACSC management in health care systems. Our goal was to present an analytical method intended to enable large health care systems to identify and prioritize actions to reduce health disparities among patients. The index also works as a comparison tool to assess impact of these interventions on health equity.

## The Health Equity Index

### What is the index?

Our index is a flexible metric that utilizes multiple data sources to quantify any excess in actual hospital encounters compared with what would be expected if health equity was realized. At Sutter Health, the index is calculated monthly for each of the 22 hospitals and disseminated through a dashboard to provide administrators with an assessment tool and starting point for evaluating disparities. Focusing on a given disease and time period, patients are segmented by age (3 groups), race/ethnicity (4 categories), and gender, and a health equity score (HES) is derived for each of the 24 groups by calculating the observed-to-expected ratio of hospital encounters. Providing a summary of the magnitude of disparities among all 24 groups, the index is calculated by taking the average, weighted by encounter frequency, of the amount by which each HES exceeds one. The index provides a summary that can be benchmarked and used as a “stoplight tool” to identify the existence of inequity among subgroups. The HES, used in calculating the index, further identifies the specific groups whose outcomes are negatively affected. For both the index and the HES, values >1.0 indicate a potential opportunity to improve the health outcomes for specific segments of the patient population.

**FIG. 1.**
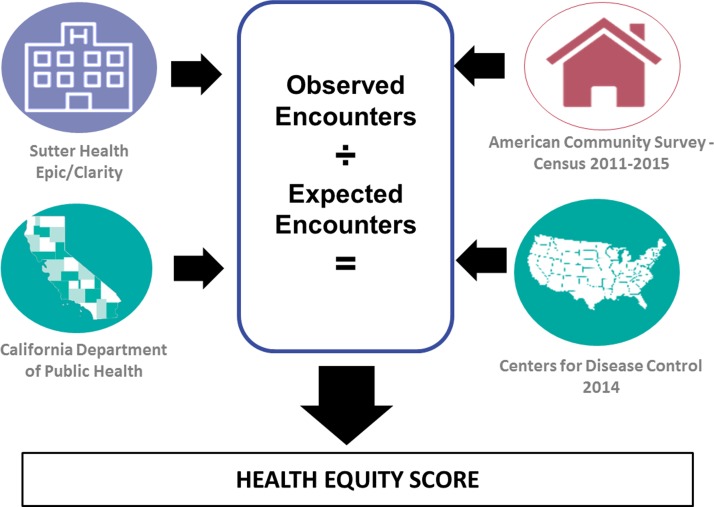
Data sources for HES. HES, health equity score.

Utilizing multiple data sources (Fig. 1), the number of expected encounters for each age/gender/race stratum is derived from the underlying population distribution within the hospital catchment area, condition prevalence, the average propensity to utilize the given hospital, and frequency of that utilization. After identifying the hospital catchment area (defined as the collection of census tracts whose residents comprise at least 80% of the hospital's patient population), underlying United States Census tract population estimates are derived from the American Community Survey and combined with published prevalence estimates to approximate the number of individuals with the given condition within the catchment area. In our calculations, we used prevalence estimates from various sources, relying on the most granular and most accurate public data available. For asthma, prevalence estimates were provided by the California Behavioral Risk Factor Surveillance System and the California Health Interview Survey and compiled by the California Department of Public Health.^17,18^ For diabetes, these estimates were taken from the Centers for Disease Control and Prevention (CDC) published Morbidity and Mortality Weekly Report.^19^ Hospital utilization is calculated from Sutter Health's EHR and used to estimate the average proportion of people with the particular condition who sought care from the catchment area and the average number of encounters made per person. The expected number of condition-specific encounters is then calculated by the product of these factors. For a more detailed explanation of the calculations, please see Supplementary Appendix 1.

The observed number of condition-specific encounters is derived directly from Sutter's EHR database. The HES for each category is defined as the ratio of observed-to-expected values, whereas the index value is calculated as a weighted average as described previously. Critical success factors for the calculation and use of the index include access to robust, self-identified race and ethnicity data, publicly available prevalence and demographic data, and the ability to extract real-time data from an EHR.

This project does not meet the definition of human subject research or clinical investigation at Sutter Health, as such Internal Review Board approval is not required.

### Why was it developed?

We first developed an index for asthma and diabetes. As an example, we present the results for asthma from one Sutter Health hospital in Alameda County, California—Alta Bates Summit Medical Center (ABSMC). In 2016, at ABSMC there were 649 patients who utilized the emergency department (ED) 877 times for asthma. The index value for this facility was 1.5 indicating that among age–gender–race subgroups that experienced higher than expected numbers of encounters, there was a 50% weighted average excess. This was primarily driven by disproportionate utilization by African American (AA) patients, and in particular, AA women 60+ years of age (HES=2.3) and AA men 45–64 years (HES=2.2). At this facility, Hispanics and non-Hispanic whites utilized emergency services less than expected across all ages regardless of gender.

Based on this information, administrative and clinical leaders at ABSMC set out to design and implement a pilot program to address the disparate outcomes for AA patients with asthma in that facility. Partnership with a community-based federally qualified health center was essential to collaboratively design and implement a program intended to address the specific needs of AAs who utilize the ED for asthma. The goals of the program are to connect patients with culturally appropriate community-based primary care, to provide education about disease and medication self-management, and to use both high-touch and high-tech solutions to provide real-time counseling services. Although the pilot is ongoing, the approach has shown the potential to help patients better manage their asthma in the ambulatory setting and avoid reliance on the use of emergency services.

Sutter Health created a steering committee, led by the Chief Medical Officer, to evaluate health equity at Sutter Health, and to assess the role of the index in improving health equity within our health system. Leadership at Sutter Health, including executive leaders and the board of directors, are committed to identify, quantify, and address inequities in health outcomes wherever they might exist. This work is a component of our quality program and part of our continuous improvement journey. The hope is that we can demonstrate the ability of provider organizations to take actions that can make a national impact on health equity. The index is a first step in helping to identify and quantify the collective impact that we, as providers, can have.

### How can the index be used?

Sutter Health is a large health system in Northern California comprising 24 hospitals (22 with emergency departments) and 5 affiliated medical foundations. Sutter provides acute and ambulatory care to >3 million people per year from >100 communities across 22 counties in Northern California. Because geography and demographics vary for each hospital, we developed health equity statistics referable to each hospital, which can also be combined into a system-wide metric. The index values for each condition are published monthly on a leadership dashboard, and a team of analysts, with expertise in health equity, is available to operational leaders for consultation. Early in 2017, the index was added as a new regular entry in Sutter's Acute Care Quality Dashboard. As part of a program to advance health equity at Sutter Health, a Health Equity Strategy Leadership Team was formed, and this team works with leadership at each hospital to help develop appropriate programs to bring equity to the patients they care for by identifying novel and unique opportunities to intervene.

### Application in an integrated health system: an example

**FIG. 2.**
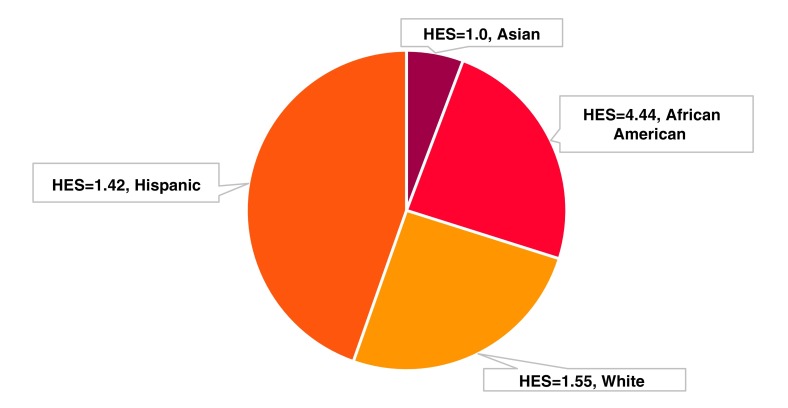
Example of HESs by race. Pie slices are proportionate to the number of encounters for each group. The health equity index is a composite measure weighted by encounter frequency.

**FIG. 3.**
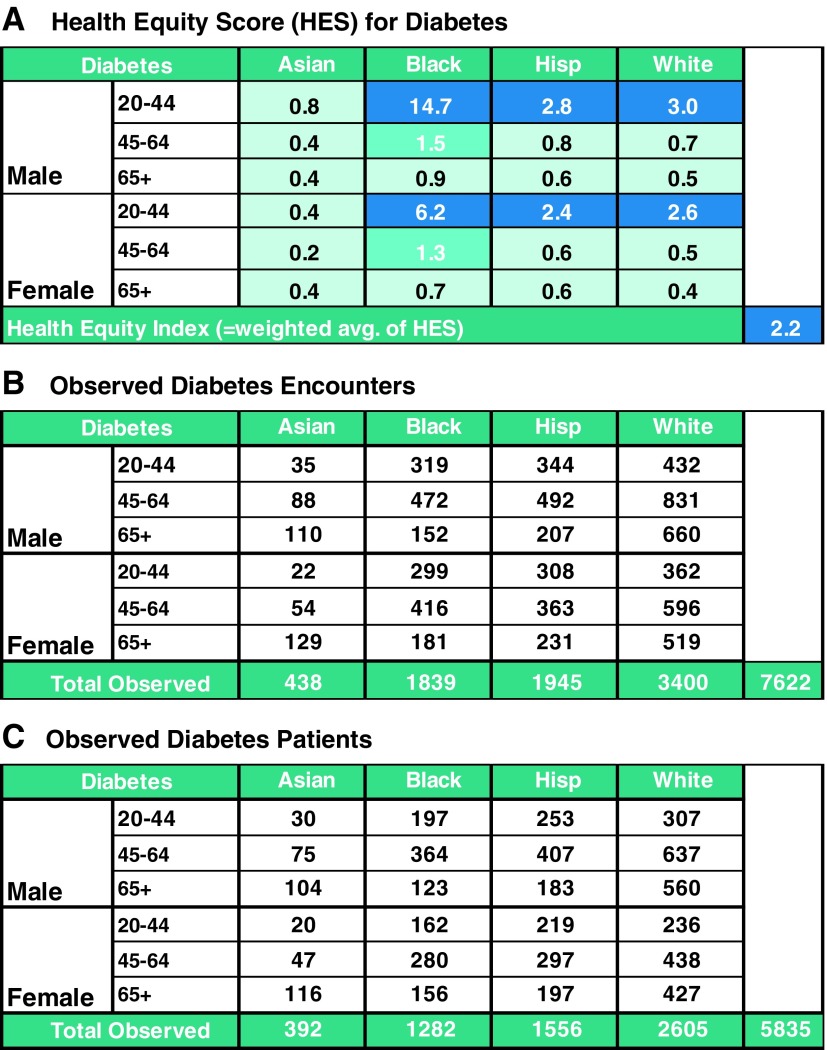
Example of HESs and relationship to health equity index. This figure consists of three different panels labeled. **(A)** HES for diabetes, **(B)** observed diabetes encounters, and **(C)** observed diabetes patients. These three panels together make up Figure 3.

In March 2017, the system-level index value for diabetes was 2.2. Breaking it down by race, it is clear that the AA patients account for approximately a quarter of the encounters, and have the highest score (Fig. 2). To further understand where the excess in observed visits for diabetes originated, the leadership team considered the HES for each gender, age, and race/ethnicity category (Fig. 3, panel A). Across AA, Hispanic, and white patients between the age of 20 and 44 years, each HES exceeded 2.0 with the highest among AA men (HES=14.7) and AA women (HES=6.2). This means that there were nearly 15 times and 6 times more encounters than expected given the underlying prevalence for diabetes among the age, race, and gender subgroups, respectively. Upon further examination of the underlying patient distributions (Fig. 3, panel B, C) they found that 197 young AA men accounted for 319 encounters and 162 young AA women accounted for 299 encounters. In total, 359 patients were not receiving the care they needed to successfully control their diabetes. As next steps, the leadership team recommended monitoring changes in the index scores over time and focusing resources on addressing potential inequalities among AAs of young age.

### How to obtain

For more information or to request an Excel version of the index code, please email Alice Pressman (pressmar@sutterhealth.org).

## Discussion

The index is a novel empirical approach to identify and quantify health inequalities in health care systems. We describe its development and provide two examples of its application at Sutter Health, a large integrated health care system. We believe this measurement system holds promise in helping to identify health inequities among patient populations served by a hospital or health care system and enables provider organizations to assess the effectiveness of interventions.

To our knowledge, this is the first attempt to develop and implement a metric for measuring health equity that uses not only real-time, health system data, but also combines it with external demographic, prevalence, and utilization statistics to compute values that reflect equity of outcomes specific to each racial or ethnic group studied. Note that the index does not rely upon normalization to any specific reference group (e.g., non-Hispanic whites). Rather, each group is compared with an internal standard, providing the ability to identify any group that may be disadvantaged and eliminating any preconceived notions about which group may experience preferred outcomes. Other attempts have been made to measure and monitor health equity. One effort, aimed at addressing disparities in primary care clinics in Baltimore (MD) used data from the Johns Hopkins Community Physician primary care EHR.^12,13^ Data elements, specifically related to patients with hypertension, were displayed on a virtual dashboard for primary care providers to see. The primary goal was to increase awareness about existing disparities. The initiative was introduced as part of a three-part effort to improve health outcomes. However, the dashboard metrics were focused on individual providers' outcomes as opposed to system-wide/hospital-wide outcomes. An approach focused on individual provider outcomes may face resistance or considerable challenges given the sensitive nature of these metrics and the implications the findings may have in terms of provider practices. Although individual providers can make constructive contributions to health equity within their practices, a system, through community-based partnerships and deployment of resources, can exert far greater leverage in changing disparate outcomes than an individual provider. Another method that has been explored by others is a health equity “audit” where data are used from EHRs within a practice setting to generate periodic health equity reports to be reviewed by the individual practice groups.^16^ The audits focus on three ACSCs; coronary heart disease, type 2 diabetes, and chronic obstructive pulmonary disease (COPD). Disparities of interest include race, age, and gender. However, there are differences compared with our proposed metric. The reports do not reflect real-time data and report the proportion of patients meeting a healthy guideline for various conditions as opposed to hospital encounters. Badrick et al.^16^ discussed the impact their reports had on the health system and report that disparities were identified, but even after surveillance and some evidence of improvement in chronic disease management, these discrepancies persist.

Recently, in England, the National Health Service (NHS) has attempted to quantify health equity by comparing preventable hospitalizations for ACSCs.^15^ Data are pulled from the NHS Hospital Episode Statistics and presented as standardized emergency admission rates versus a neighborhood deprivation rank. However, in contrast to our proposed metric, the data are presented as a one-time report to be delivered to management rather than a real-time, actively updated dashboard. Reports and analysis are focused on the national level in England as opposed to a system or site level. In addition, this initiative does not examine race/ethnicity, but instead focuses on differences between neighborhoods.

Another major difference between previously reported health equity measures and our index is that ours is designed to be portable to any health care system. The only requirements are access to health records, including encounter diagnoses, geographical data, age, gender, and race/ethnicity; access to local census tract data; and access to local-level prevalence data. The index is also designed to be adapted to any health condition as long as there are available prevalence data.

The index presented here is not intended to replace traditional methods of identification of disparities. Rather it should provide a different vantage point. In developing and vetting the index, we compared results with those obtained through our standard methods of identification of disparities; comparison of rates by racial/ethnic subgroup with white serving as the standard. We found that in general if the inequity was very large, the methods were similar; however, with the traditional methods that do not adjust for underlying population characteristics, where a subgroup is overrepresented in a geographical region, the inequity may be magnified. Conversely, where a subgroup is underrepresented, standard methods may mask smaller inequities. In addition, our index can be used to identify gender and age inequity, typically more difficult to discern because there is no obvious reference group.

There are several limitations to the use of this index. First, the calculation of “expected counts” is constrained by availability of prevalence data for the underlying condition. For asthma and diabetes, we have used publicly available state-wide data from the California Department of Public Health and the CDC, but we have not yet identified a source for COPD and heart failure, the next two high-priority ACSCs for our health system. For the prevalence estimates, the closer the denominator's geographic unit is to the underlying catchment area, the more accurate the “expected” calculations will be. Second, users must have access to adequate sources of EHR data from which to derive “observed” counts. We are fortunate that the Sutter Health EHR is comprehensive and integrated across all hospitals in the system. Finally, in the process of calculating “expected counts,” we have made several assumptions: the prevalence of the condition in our system is similar to those values for the state of California; the demographics of the census tracts are similar to our patients from those tracts who utilize our EDs; the prevalence estimates for each subgroup can be applied uniformly across everyone in that subgroup.

It is the authors' hope that the index will be widely implemented and tested by others and adapted for additional health conditions. It also has the potential to serve as a prototype for the development of future metrics that combine an even greater degree of patient (consumer) data that can yield greater precision in identifying inequities. As providers, the extent to which we can understand inequities with greater precision allows us to craft more targeted and hence more effective solutions.

## Conclusion

The index is a novel, innovative metric that provides the information to allow a health system to identify population subgroups that suffer the most from outcome disparities, and to develop interventions to address these inequities. This tool facilitates the targeting of interventions to particular care facilities where minority patients are likely to receive most of their care. Identifying and measuring health outcome disparity is the first step toward achieving health equity, and it will allow us to turn our efforts toward understanding and correcting the underlying causes.

## Supplementary Material

Supplemental data

## Abbreviations Used

AA: African American
ABSMC: Alta Bates Summit Medical Center
ACSC: ambulatory care sensitive condition
CDC: Centers for Disease Control and Prevention
COPD: chronic obstructive pulmonary disease
EHR: electronic health record
HES: health equity score
NHS: National Health Service
