# Supplementary material for: Measuring Health Equity for Ambulatory Care Sensitive Conditions in a Large Integrated Health Care System: The Development of an Index
Source: Health Equity. 2019 Apr 3;3(1):92–8. doi: 10.1089/heq.2018.0092 (PMC6450454; doi:10.1089/heq.2018.0092)
Supplement: Supplemental data [file Supp_Appendix.pdf]

Appendix 1. Detailed underlying calculations for asthma example

Health Equity Index for Ambulatory Care Sensitive Condition (ACSC) Hospitalizations

Definition

The Sutter Health Equity Index score is the maximum of the individual index scores for 4 Ambulatory Care Sensitive Conditions (ACSC): Diabetes (DM2), Asthma, Chronic Obstructive Pulmonary Disease (COPD), and Congestive Heart Failure (CHF). The individual index score for each ACSC is in turn calculated as the average excess (>1) of Health Equity Scores (HES's) for each race/ethnicity category weighted by the total number of encounters for that group. Each HES is calculated as the ratio of *observed* to *expected* hospital admissions (including ED and inpatient), adjusted for age-, sex-, race/ethnicity-specific prevalence of the condition, propensity of the subgroup to utilize a particular hospital, and the underlying catchment-population distributions.

Data Sources

Hospital admissions: Epic/Clarity/Midas
Population distribution: US Census American Community Survey 2015 5-yr estimates
Prevalence estimates:

DM2: Centers for Disease Control 2014 chronic disease publication

Asthma: the California Behavioral Risk Factor Surveillance System and the California Health Interview Survey and compiled by the California Department of Public Health.

COPD: TBD

CHF: TBD

Benchmark goal source: Sutter Health Advancing Health Equity Dashboard

Measurement Calculation

Health Equity Score (HES_i)
Note: these are based on 3 age groups, 2 sexes, and 4 racial/ethnic groups (NHW, NHB, NH, and Hispanic) - there are 24 categories *i*, 1-24.

Numerator: The *observed* number of total hospitalizations (encounters) for given condition age, sex and racial/ethnicity group. Only primary Dx is counted.

Denominator: Total *expected* number of hospitalizations for given condition age, sex and racial/ethnicity group. Expected values are derived as follows:

1. Identify catchment area by determining those census tracts that account for 80% of all hospital encounters (sort by counts from each tract and include top tracts until reach 80% of encounters)
2. Determine the actual age/sex/race population distribution of the catchment area – from publically available US Census data)
3. Using prevalence estimate from the smallest geographical unit available (we used State-level), apply the prevalence rates to estimate number of cases of ACSC in the catchment area.
4. Calculate proportion of people with ACSC in the entire catchment area who utilize the particular hospital. Call this the rate of utilization.
5. Assume that if there was no difference by age/sex/race then each age/sex/race group would utilize at the same rate, and apply this rate of utilization to the underlying estimated number of cases of the ACSC to determine expected numbers of utilizers.
6. Calculate the expected number of encounters per person (using total encounters for any reason) in each category.
7. Apply the expected number of encounter per person to expected number of ACSC utilizers to get expected number of encounters in each category.

$Expected_i$ = (Overall rate of utilization for catchment area)*(expected number of cases of ACSC in subgroup i)*(encounters per person in i)

HES_i = *Observed ACSC* $Encounters_i$ / *Expected ACSC* $Encounters_i$

where i is the age/sex/race category (i=1-24)

Average excess (Index)

1. For all values >1, the excess is weighted by observed total encounters in each subgroup i
2. The weighted average of these excesses is added to 1.
3. Calculation: $1+\left\{\sum_{i=1}^{24}\left[\frac{(HES_i-1)(Encounters_i)}{(Total\ Encounters)}\right]\right\}$

This methodology can be extended to calculate an index (I) for each racial category (i.e. I_W, I_{ASIAN}, I_{AA}, I_{HISP}) to identify the areas of opportunity and to monitor hospital progress.

Finally, a single overall dashboard metric can be calculated as follows:
Maximum I = Max[I_{DM2}, I_{CHF}, I_{COPD}, I_{ASTHMA}]

Metric

The Index is the average excess health equity score for each Ambulatory CareSensitive Condition Hospitalizations for a rolling 12 month period.
Lower I’s that are equal to or less than 1.0 are better.

Measure Target & Threshold

All I’s are equal to or less than 1.0.
